# Psychiatry must not be separated from its historical and cultural context

**DOI:** 10.4103/0019-5545.31599

**Published:** 2006

**Authors:** Paul Hoff

**Affiliations:** * Professor of Psychiatry University of Zurich, Department of General and Social Psychiatry, Lenggstrasse 31, P.O. Box 1931, CH 8032 Zurich, Switzerland e-mail: paul.hoff@puk.zh.ch, www.pukzh.ch

A.R. Basu's call for a proper implementation of the historical and cultural perspective in research on Indian psychiatry and its history is highly justified.^1^ This commentary will mainly reinforce his view. Initially, I shall say a few words about the practical relevance of the history of psychiatry in general, given the present-day debate on psychiatry's identity (I). This will be in line with arguments published previously.^2^ Then I shall mention one additional argument concerning the theoretical foundation of psychiatry (II.1) and two debatable issues with regard to the author's position (II.2) before concluding with four theses (III).

## I. HISTORY OF PSYCHIATRY IS PRACTICALLY RELEVANT

The main hypothesis of Basu's paper is that the understanding of the history of psychiatry in India has for a long time been too strongly linked to two western phenomena. These are—on the theoretical side—the *project of enlightenment,* and—on the political side—*colonialism*. The author is critical of the global claims of enlightenment ideas, especially the notion of rationalism being the only proper way to develop democratic societies, thus pushing every ‘irrational’ moment aside or even declaring it dangerous or insane. And he is critical of the willingness of Indian psychiatry to shape its self-under-standing according to the concept and practice of British psychiatry which was brought to—perhaps forced upon—India in colonial times.

What has this to do with the practical relevance of research into the history of psychiatry? First of all, the history of psychiatry is a very heterogeneous field. It consists of, or at least deals with, many different, often conflicting scientific cultures and traditions. Nonetheless, the following four arguments should be considered:

### • The *historical* argument

We need the history of psychiatry to collect documents and other historical sources on authors, concepts and institutions of psychiatry, and to understand their position within the different scientific traditions of psychiatry.

### • The *practical* argument

It is not only a theoretical issue to deal with the history of psychiatry, but also a highly practical one. This has to do with the fact that ‘mental illness’, whatever definition one might apply, will never be just one self-explanatory concept. Different approaches to define ‘mental illness’ have a significant impact on diagnosis and therapy, e.g. the several controversial concepts on ‘borderline states’ end up with completely different diagnostic procedures (operationalized v. heuristic) and therapeutic options (interpretation of conflicting personality structures and their development since childhood v. skills training v. mood-stabilizing or other psychotropic drugs). Of course, modern psychiatry increasingly tries to integrate different approaches in order to find the most effective treatment for the individual patient. But the point is that there are indeed significantly different and practically relevant ways to conceptualize major psychiatric issues. And to adequately understand these complex and long-standing developments, we strongly depend on the historical dimension.

### • The *theoretical* argument

This argument is called theoretical because it refers to the risk of any given psychiatric theory to become uncritical, ‘narrow-minded’ and—in the worst case—dogmatic. If one looks closely at the history of our field it becomes evident that dogmatic positions in fact did occur in every psychiatric line of thought. As discussed elsewhere in greater detail, there are at least three major concepts of mental illness:^3^ The biological or naturalistic one (‘mental illness is a brain disease’), the psychological or heuristical one (‘mental illness is an understandable reaction or development within the patient's biography’) and the nominalistic one (‘mental illness can at present not be sufficiently defined as a real object, e.g. as a brain disease; however, we can develop operationalized criteria for the terms we use to *describe* mental illness, such as schizophrenia—the ICD-10/ DSM-IV approach). Each of these different ways of understanding mental illness may evolve into a dogma. The biological or, better, neuroscience approach could turn into ‘brain mythology’, the psychological and heuristic perspective into ‘psychologism’ and social psychiatry into some kind of ‘social mythology’, if these methods are not carefully applied according not only to their possibilities and advances, but also to their limitations. The key message in our context is—detection and prevention of psychiatric myths also strongly depend on historical and cultural knowledge.

### • The *political* argument

Psychiatry has a special responsibility not only towards patients, but also towards society. For example, no other medical specialty is so often involved with involuntary admissions and treatments as is psychiatry, no other medical specialty has such a close and complex relationship to jurisprudence as (forensic) psychiatry. To adequately meet the demands arising from this special situation, psychiatry cannot do without the historical and theoretical (especially epistemological) perspective and the notion of personal autonomy.

## II.1. THE DANGER OF ACCEPTING FRAMEWORKS THAT ARE TOO NARROW

It is very plausible that from the Indian perspective the ‘colonial attitude’ of interpreting the history of psychiatry plays a crucial role in this debate. But it would be a mistake to believe that in other regions of the world there is no risk of influencing psychiatric concepts by more or less implicit, ‘tacit’ philosophy, which sometimes turns into plain ideology. Or, to put it the other way round, the need to connect psychiatry with its historical and cultural roots is present all over the world. In the past two or three decades, the biological or naturalistic paradigm has become *the* point of reference in psychiatric research, given the fast and impressive gain in the knowledge of brain function in the case of mental health and mental disorders. But, as has been often demonstrated, this neuroscientific approach to psychiatric practice and research *may* develop (not necessarily, of course!) into an unreflected reductionistic naturalism. Eliminative materialism is the most prominent example of this. And here we also find a tendency to significantly underestimate the theoretical, historical and cultural underpinnings of *any* psychiatric theory. To put it more decisively, any psychiatric concept—be it biological, psychological or sociological in nature—is basically at risk for developing into an uncritical and unhistorical dogma, sometimes even proudly (and falsely) announced as ‘atheoretical’.

## II.2. TWO DEBATABLE ISSUES

Regarding two aspects, the author's critique might go a little far:

First, the valuation of Foucault's work on psychiatry seems too enthusiastic. Of course, Foucault—like many authors of the ‘anti-psychiatry’ movement in the 1960s and 1970s—induced a sharp and fruitful debate on psychiatry's relation to society. But, his view also had its shortcomings, e.g. when interpreting psychiatry nearly exclusively in the light of the seclusion and labelling approach, thus underestimating the personal tragedy that the occurrence of (severe) mental illness often means to the people affected and to their relatives and friends.

Second, it is perfectly all right to draw attention to the fact that—like any other theory—the project of enlightenment does have its limitations, e.g. the issue of overemphasizing the rational and underrating the affective-volitional aspects of human mental life and behaviour. But by doing so, one should not forget the significant positive impact of the notion of subjectivity and personal responsibility on the development of modern democratic societies. It would end up with a grossly reduced understanding if we would identify enlightenment with rationalism. The Kantian approach, for example, reaches far beyond rationalism.

Hence, in my view, psychiatry should adopt an undogmatical combination of central ideas of the enlightenment project—especially personal autonomy—with the historical and cultural perspective. None of these three perspectives must be omitted, since in that case the following consequences might result:

- By omitting the *enlightenment project,* the notion of personal autonomy will be at risk, especially with regard to the autonomy, which a person still has—and indispensably has—even in the presence of a mental disorder. The worst case here would be the acceptance of an uncritical reductionism of whatever type (e.g. on the grounds of biology, psychology or sociology). Such a reductionism would be harmful not only to the self-understanding of persons, but also to our attempts to create and apply an epistemologically informed framework for scientific research on mental and social phenomena.
- By omitting the *historical perspective,* the emergence of modern psychiatric concepts will not be sufficiently understandable. We will then not be able to learn—both in a positive and negative sense—from the arguments brought forward by the founders of our field, e.g. Wilhelm Griesinger, Emil Kraepelin, Eugen Bleuler or Karl Jaspers.
- By omitting the *cultural aspects,* we ignore the multitude of human approaches to life and society in general and to mental illness in particular in an unscientific manner, thus ultimately even giving way for a subtle kind of scientific neo-colonialism.

## III. CONCLUSION: FOUR THESES

1. The history of psychiatry is not ‘l’art pour l'art’. It is of crucial importance for the understanding and development of theoretical and practical issues in our field.
2. Research on the history of psychiatry is necessarily an interdisciplinary and international process.
3. The global perspective is highly important, but it will never be able to replace genuine locally-based research. But this is also true the other way round: Research on specific local, regional or national issues will be (and should be) signi-ficantly enriched by an international and intercultural approach.
4. Psychiatry's self-understanding is, at present, challenged by a couple of overt or implicit, one could say ‘tacit’, uncritical presuppositions or, more straight forward, prejudices. The political prejudice, for example, is at work, when the history of psychiatry in India is told *only* as the story of successful western concepts replacing ‘irrational’ local traditions that are not even regarded suitable for thorough historical research. Another major type of prejudice, the theoretical one, supposes that only one scientific approach, e.g. naturalism, may serve as relevant framework for the whole field. Therefore, one distinguished assignment for the history of psychiatry is to detect and, ideally, minimize the effects of prejudice by elucidating the conceptual history of the field.

## References

[1] Basu AR (2005). Historicizing Indian psychiatry [Viewpoint]. Indian J Psychiatry.

[2] Hoff P, Christodoulou GN (2005). Recent advances in research on the history of psychiatry. Chances and limitations of the global perspective. Advances in psychiatry.

[3] Hermanni F, Buchheim Th, Hoff P. Leib & Seele, Gehirn & Geist, Gesundheit & Krankheit (2006). Die Psychiatrie als Schnittstelle medizinischer, philosophischer und gesellschaftlicher Kontroversen. Das Leib-Seele-Problem. Antwortversuche aus medizinisch-naturwissenschaftlicher, philosophischer und theologischer Sicht.

